# Uncovering the Pathogenic Landscape of Helminth (*Opisthorchis viverrini*) Infections: A Cross-Sectional Study on Contributions of Physical and Social Environment and Healthcare Interventions

**DOI:** 10.1371/journal.pntd.0005175

**Published:** 2016-12-07

**Authors:** Xueyuan Ong, Yi-Chen Wang, Paiboon Sithithaworn, Jutamas Namsanor, David Taylor, Luxana Laithavewat

**Affiliations:** 1 Department of Geography, National University of Singapore, Singapore; 2 Department of Geography, National University of Singapore, Singapore; 3 Department of Parasitology, Khon Kaen University, Thailand; 4 Department of Parasitology, Khon Kaen University, Thailand; 5 Department of Geography, National University of Singapore, Singapore; 6 Disease Prevention and Control 7, Khon Kaen, Thailand; University of Florida, UNITED STATES

## Abstract

**Background:**

Helminth infections have proven recalcitrant to control by chemotherapy in many parts of Southeast Asia and indeed farther afield. This study isolates and examines the influence of different aspects of the physical and social environment, and uneven intervention effort contributing to the pathogenic landscape of human *Opisthorchis viverrini* infections.

**Methodology:**

A cross-sectional survey, involving 632 participants, was conducted in four villages in northeast Thailand to examine the impact on prevalence and parasite burden of the reservoir dam environment, socio-economic, demographic, and behavioral factors, and health center intervention efforts. Formalin-ether concentration technique was used for diagnoses, and multivariate models were used for analyses.

**Principal Findings:**

The importance attributed to *O*. *viverrini* infections varied among health centers in the four study villages. Villages where *O*. *viverrini* infections were not prioritized by the health centers as the healthcare focus were at a higher risk of infection (prevalence) with odds ratio (risk factor) of 5.73 (3.32–10.27) and p-value < 0.01. Priority of healthcare focus, however, did not appear to influence behavior, as the consumption of raw fish, the main source of *O*. *viverrini* infections in the study area, was 11.4% higher in villages that prioritized *O*. *viverrini* infections than those that did not (p-value = 0.01). Landscape variation, notably proximity to reservoir, affects vulnerability of local population to infection. Infection intensity was higher in population located closer to the reservoir with risk ratio of 2.09 (1.12–4.02) and p-value < 0.01. Patterns of infection intensities among humans were found to match fish infection intensity, where higher infection intensities were associated with fish obtained from the reservoir waterbody type (p-value = 0.023).

**Conclusions/Significance:**

This study demonstrated the importance of environmental influence and healthcare focus as risk factors of infections in addition to the socio-economic, demographic, and behavioral factors commonly explored in existing studies. The reservoir was identified as a crucial source to target for opisthorchiasis intervention efforts and the need to consider infection intensity in disease control efforts was highlighted. The holistic approach in this study, which underscores the close relationship between the environment, animals, and humans in development of human infections or diseases, is an important contribution to the framework of One Health approach, where consideration of helminth diseases has largely been overlooked.

## Author Summary

Many of the large-scale helminth control programs around the world have primarily relied upon drug treatment. Reliance on drug treatment alone does not deal with the ultimate causes of infection, resulting in reduced infection levels only in the short-term. Re-emergence of infections and possibly even development of drug resistance in parasites are common once the programs have been terminated. There is thus a need for consideration of a broader context, including environmental influence and healthcare focus, within which infections thrive. This study examines the roles of a reservoir dam environment, inter-provincial healthcare focus variation, and socio-economic, demographic, and behavioral factors to highlight the varying roles of such factors contributing to this disease landscape. The findings underscore the importance of a holistic approach in infection studies in order to provide more sustainable disease treatment and elimination outcomes.

## Introduction

Helminthiases, which include foodborne trematodiases, lymphatic filariasis, schistosomiasis, and soil-transmitted helminthiases, are the most common neglected tropical diseases (NTDs) in southeast Asia [1]. They disproportionally affect the poor or marginalized population in developing countries, trapping the afflicted in a vicious cycle of poor health outcomes and poverty, and costing billions of dollars in treatment each year [2]. The increasing recognition of the burden caused by helminth infections has brought about large-scale control programs by the World Health Organization and other nationwide control programs in countries in Asia [3,4], Latin America [5], and sub-Saharan Africa [6], where helminthiases are prevalent. These programs have primarily relied upon chemotherapy for helminthiases control [7].

Many chemotherapy programs have relatively limited objectives, resulting in reduced infection levels in the short-term [8]. Re-emergence of the disease, and possibly even development of resistant strains of parasites, is common once a program has been terminated, however [7]. Evidence already exists of the reduced efficacy of drugs used to combat lymphatic filariasis [9] and schistosomiasis [10], with frequent treatment involving anthelmintic drugs appearing to hasten the development of drug resistance in some animals [11]. While chemotherapy has reduced levels of infection in the short-term, ensuring that positive health benefits extend beyond the cessation of chemotherapy programs has been challenging without improvements in the other factors that predispose populations to helminthiases [12,13]. Helminth infections, and indeed many infectious diseases, are strongly influenced by environmental and socio-economic conditions, and by human behavior and the effectiveness of health service provision [13], or what Lambin et al [14] term the pathogenic landscape for disease. A major increase in schistosomiasis following the construction of dams and irrigation infrastructure has been well-documented [15], as has the eradication of schistosomiasis in Japan through modernization of agricultural practices [16] and reduced hookworm infections as a result of improvements in sanitation and housing [17].

A One Health approach permits consideration of vulnerabilities at the environment-animals-humans interface [18,19], accounting for the complex and highly dynamic process of infection, where a change in one underlying factor can drastically alter the situation for the other conditions, leading to the uneven distribution of diseases even in places with seemingly similar conditions. Such unevenness is observed in opisthorchiasis, an infection caused by the foodborne trematode *Opisthorchis viverrini*, where large variations in the disease burden may be observed in a relatively small geographic area [20,21]. Despite the close association of helminth parasite life cycle and life strategies with the physical environment and animal hosts, the One Health approach has rarely been applied to study of helminthiasis [22]. Yet, understanding such factors that underpin infections with a high focality can provide important contributions to the framework of One Health approach to a broader range of diseases, enabling intervention efforts that are tailored to local pathogenic landscapes, and in particular finely resolved vulnerabilities to the disease, to better accommodate future variations [23]. Moreover, the influence of, for example, environmental conditions can be easily masked by other factors that contribute to the extent and severity of a disease outbreak, such as health intervention efforts [24]. For example, intensive chemotherapy efforts have mitigated schistosomiasis burdens associated with recent hydro-infrastructure developments [25,26], but such effects are palliative and temporary if the underlying factors causing infections, including infection of animal hosts and environmental conditions that promote and maintain pathogenesis, remain [27].

Opisthorchiasis is a major NTD in southeast Asia, and in the Mekong River basin in particular. The parasite involved, *O*. *viverrini*, is one of only three metazoan pathogens classified as a group 1 carcinogen, with sufficient evidence to establish a link between *O*. *viverrini* and cancer in humans [28]. Carcinogenicity of opisthorchiasis stems not only from prolonged infection and re-infection but also from the repeated treatment involving praziquantel anthelminthic, which can induce DNA damage leading to the development of hepatobiliary abnormalities, including cholangiocarcinoma (CCA) [29,30]. CCA is among the leading causes of cancer-associated mortality in the Mekong River basin [31].

*O*. *viverrini* is closely associated with wetland (rice)-based agriculture where drainage canals can facilitate infection of fish hosts by snail-shed cercariae [32]. The trematode has a three-host life cycle with freshwater *Bithynia* spp. snails and cyprinid fish as, respectively, the first and second intermediate hosts, and humans as the definitive host [33]. Human infection occurs through the consumption of raw or undercooked cyprinid fish, which is a common practice in the Mekong River basin. Small-scale freshwater fishing activities provide a major source of protein and additional income for local communities [21], while raw fish consumption has led to the persistence of opisthorchiasis in many parts of the region despite decades of control efforts [34,35]. The control efforts have, to date, largely been restricted to chemotherapy and education campaigns, where the measure of success of control programs is limited to prevalence reduction instead of reinfection rate and long-term sustainability [36]. Despite the close relationship of opisthorchiasis with the physical and social environment, research on the range of factors that underpin the cycle of infection and reinfection has largely been neglected [18,37]. Particularly, there is little information on the association between human infection intensity and fish infection variation in different waterbody types. In fact, infection intensity is much less frequently reported than infection prevalence in *O*. *viverrini* studies [eg. 38–40]. The same is the case for other helminthiases, including soil-transmitted helminthiases [41]. This is problematic because infection intensity enables a very different understanding of the disease transmission and life strategies as compared with infection prevalence, in addition to being a factor in the most severe forms of infectious disease, including the risk of developing CCA in the case of *O*. *viverrini* infections.

The focus of this study is the pathogenic landscape for opisthorchiasis, in particular the epidemiological role of dam construction and subsequent reservoir creation, socio-economic conditions, demographic factors and behavior, and variations in the efficacy of the provision of health services. This study illustrates the causes of an uneven distribution of disease burden, identifying contributing factors of infection while controlling for existing chemotherapy control efforts. Infection intensity is determined in addition to infection prevalence, and the variations in factors shaping intensity and prevalence examined. This study has the potential to facilitate improved health intervention efforts that take into account the high focality of opisthorchiasis. The approach and results have wider applicability, to the study of other NTDs, especially those with complex, environmentally sensitive life cycles.

## Methods

### Ethics statement

Ethical approval for this study was obtained from the institutional review board of National University of Singapore, Singapore (Reference code: A-14-122, approved on 20 August 2014) and Khon Kaen University, Thailand (Reference code: HE571229, approved on 22 July 2014). Permission for fieldwork was obtained from the subdistrict health centers. Meetings were held with heads of the health centers and health center workers to explain the purpose, procedures, risks, and benefits of the study. Health center workers were briefed, using Thai language, on the participant information sheet and the need to obtain written consent from the participants, and on how to administer the questionnaire, and to obtain fecal samples. All adult subjects were informed about the study design and objectives, and all study subjects gave written consent. No children were involved in this study. Identifiable information collected including names were anonymized using code numbers. After fecal examination, for participants tested positive with parasitic infection, personal information and corresponding infection results were made available only to the health center in the village so that treatment could be administered. Deworming medication was provided to the health centers for treatment of participants who were tested positive with infection. Those infected with *O*. *viverrini* were treated with praziquantel at an oral dose of 40 mg/kg. All medications were administered by certified nurses from the health centers. After the survey, only code numbers were retained by the principle investigator with the infection results and survey responses. No identifiable information was kept nor published.

### Study area

**Fig 1:**
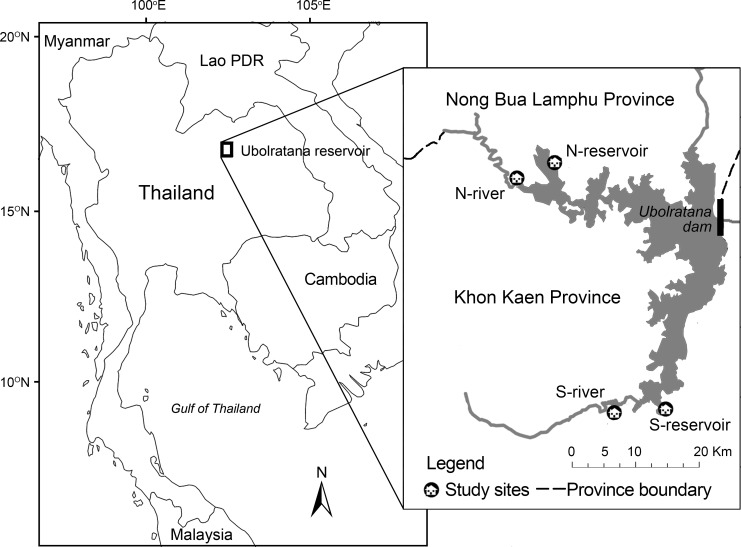
Location of study sites. Ubolratana reservoir in northeast Thailand and the four study sites, two in the north of the reservoir, and two in the south.

This study was conducted in four villages in the catchment for the Ubolratana reservoir (16°43’40°N, 102°34’45°E), northeast Thailand (Fig 1). Two of the villages, Sai Mun and Huay Bong, are located in the province of Nong Bua Lamphu, to the north of the reservoir. The other two, Fa Luem and Pho Tak, are in the province of Khon Kaen, to the south of the reservoir (Fig 1). According to Ong et al [42], levels of *O*. *viverrini* infection of intermediate fish hosts were greater in fish caught in the main body of the reservoir when compared with those captured in rivers draining into the reservoir. In order to examine the influence of the physical environment on human *O*. *viverrini* infection, villages of varied levels of exposure to fish infected with *O*. *viverrini* were sampled. Two of the villages sampled, one in the north and one in the south of the reservoir, are located along the river inlets in the study area, and two, one in the north and one in the south of the reservoir, are located along the shore of the main body of the reservoir. Hereinafter, the villages are referred to as north (N)-river, N-reservoir and south (S)-river, and S-reservoir. *O*. *viverrini* infection prevalence and intensity were compared between villages located along the river inlets and reservoir to highlight and examine possible environmental influences. Samples in the north and south of the reservoir were compared to determine the association of infection with inter-provincial health jurisdiction. For reference, infection prevalence and intensity for each village were also presented, but no analyses were performed on them.

### Participants

A cross-sectional study was conducted between August and December, 2014. Fecal samples and questionnaire-based surveys on socio-economic, demographic, and behavioral factors of participants (S1 File and S2 File) were collected from August 2014, and any infected individuals identified from the results were treated during the months of November and December 2014. Participating households were selected from information provided by the local health center using a random number generator. All members from the selected households who were 21 years or older at the time of the survey were invited to participate. Using StatCalc in Epi Info 7.1.5 software at confidence interval level of 95% and margin of error at 5%, a sample size of 125 was needed for each village. A total of 756 participants from the four study villages were eventually invited.

### Determining human infection status

Current infection status involving *O*. *viverrini*, other foodborne parasites, and soil-transmitted helminths were determined from the analysis of fecal samples. A single fecal sample was provided by each participant. The samples were returned to the health center on the same day and kept on ice. Samples were transported to the laboratory the following morning where they were stored at -20°C until analyzed for their parasite content. To increase the number of fecal samples returned, each village was visited on two consecutive mornings for the transportation of samples. Samples were processed using the formalin-ether concentration technique [43] and examined under the microscope by experienced laboratory technologists. The formalin-ether concentration technique is the current gold standard diagnostic for *O*. *viverrini* infection [44], although immunological and molecular techniques to increase the sensitivity and specificity of diagnoses are being developed [34, 44]. *O*. *viverrini* eggs were counted and recorded, and evidence of other intestinal parasites noted. Infection prevalence was tabulated by dividing the number of infected people with the total number of people sampled, while infection intensity was determined as the number of *O*. *viverrini* eggs per gram (epg) of fecal sample. Infection statuses of participants were provided to the head of the health centers along with medications for the treatment of *O*. *viverrini* and other intestinal parasites. Information on past treatment of *O*. *viverrini* was obtained from both health center records and completed questionnaires (the latter were used to identify participants who received *O*. *viverrini* treatment from institutions other than health centers, including hospitals).

### Collecting socio-economic, demographic, and behavioral information

A questionnaire-based survey was conducted to determine the association of socio-economic, demographic, behavioral factors with *O*. *viverrini* infection prevalence and intensity. Variables used in this study were selected based upon existing studies on *O*. *viverrini* risk factors [45,46], while the set of possible responses in the multiple-choice questionnaire were formulated based on preliminary semi-structured interviews conducted with 251 respondents in the catchment of the Ubolratana reservoir.

Demographic information, such as age and gender, of participants were provided by the health centers. Age was tabulated based on the year of birth of the participant and was expressed as a continuous variable. Other data, including level of education and occupation, were obtained through the questionnaires. As each participant may have more than one occupation, the various occupation types were each presented as an explanatory variable. Per capita income was calculated by dividing household income by the number of household members. Participants were considered as living “Below poverty line” or “Above poverty line” by comparing their household’s per capita income to average 2014 poverty line values from the National Economic and Social Development Board of Thailand for the provinces of Nong Bua Lamphu (2357 baht) and Khon Kaen (2514 baht) [47]. Participants were given the option of whether they wished to disclose information on their income.

Levels of awareness of the hazard of *O*. *viverrini* infection and patterns of consumption of the raw fish dishes *Koi pla* (freshly prepared raw fish salad), *Mum pla*, and *Pla som* (both of which are lightly fermented raw fish dishes), which are commonly eaten in the study area, were determined through the questionnaire survey. Participants were also asked for the reasons behind their consumption/non-consumption of raw fish. Variables examined in this study were summarized in S1 Table.

### Collecting information on healthcare focus, perceptions, and chemotherapy history

The Isarn Agenda, a program aimed at CCA prevention and control in northeast Thailand, was introduced in 2012. The program involves fecal examination, ultra-sound scan for CCA above 40 years of age, exhibiting risky behavior, notably the consumption of raw fish. People found with opisthorchiasis are treated. Education programs are also created for primary school children. The Isarn agenda is not equally applied throughout northeast Thailand, however, as each province has the autonomy to decide on health priorities locally. In Khon Kaen province, in the southern part of the study area, only two districts, which are not included in this study, adopted the Isarn agenda, while other districts opted to focus on non-communicable diseases, such as cardiovascular diseases and diabetes. All districts in Nong Bua Lamphu, in the northern part of the study area, adopted the Isarn agenda. Thus, of the four villages examined in this study, the N-river and N-reservoir villages adopted the Isarn agenda, and the S-river and S-reservoir village did not.

The prevalence and intensity of infections in the four study villages were compared with past *O*. *viverrini* infection diagnostic tests conducted by the health centers. Unlike mass drug administration efforts for other helminth parasites, praziquantel anthelmintic were given as a treatment for *O*. *viverrini* only for patients who were tested positive for infection by the parasite. As such, past attempts in *O*. *viverrini* diagnostic tests can also be used to determine past chemotherapy efforts by the health centers. Furthermore, information on local health priorities and perceptions of opisthorchiasis was also obtained from the heads of the health centers.

### Statistical analysis

Criteria for inclusion in the analyses included providing consent, not having withdrawn from the study, provision of suitable stool sample, and having a completed questionnaire. The prevalence and intensity of *O*. *viverrini* infections, and the reasons for/for not consuming raw fish, were analyzed for their association with environmental factors. The most notable environmental factor included in analysis was the type of waterbody from which the fish used in raw fish dishes originated from (river or reservoir). Examined social factors included *O*. *viverrini* awareness, age, gender, and occupation. The possible influence of interventions by health centers, and variations in their level of implementation, was also investigated (S1 Table). Bivariate analyses were first performed on each explanatory variable; variables with p-values below 0.2 were next entered into multivariate models. To examine prevalence, data from all participants were used in analyses. To examine intensity, only participants who tested positive for infection were included. To examine reasons for consumption, only participants who consumed raw fish were used for the analyses; conversely, in examining reasons for non-consumption, only participants who do not consume raw fish were used. Logistic regression was employed for analyzing infection prevalence, reasons for consumption, and reasons for non-consumption. The models were simplified with backward elimination and variable deletion determined using a chi-squared test for non-significant difference in deviance. Quasi-Poisson regression was used for analyzing infection intensity in the case of overdispersion. The model was simplified with backward elimination and variable deletion determined using F-test for non-significant difference in deviance. In addition, chi-squared test was used to test for variation in proportion of raw fish consumption by location, gender, and *O*. *viverrini* awareness.

Variations in level of infections of fish by waterbody type (reservoir or river inlet) were also analyzed, using data from Ong et al [42]. A t-test with unequal variances was used on log (x+1) transformed data, as data were not normally distributed. Differences in levels of infections in fish according to waterbody type [42] are compared with results from this study. Results from these analyses were used as a basis for examining the role of factors that have contributed to the pathogenic landscape for opisthorchiasis in the study area.

## Results

### Summary of data collected

**Fig 2:**
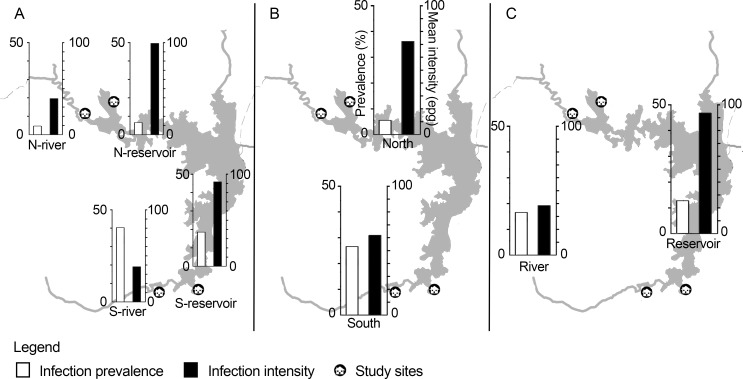
Spatial variation in *O*. *viverrini* infection prevalence and mean infection intensity. Comparison of infection prevalence and mean intensity by (A) villages, (B) provincial health jurisdictions, and (C) waterbody types.

Of the 756 participants invited, 632 suitable samples were obtained (83.60%). The mean age of participants is 52.6 years. Among the participants, 54.2% were females and 45.8% were males. Comparison of the *O*. *viverrini* infection prevalence and intensity of the four villages showed that the S-river village had the highest prevalence at 40.21%, while the N-reservoir village had the highest infection intensity at 99.41 epg (Fig 2A). When the villages were grouped according to their provincial health jurisdiction, infection prevalence in the north villages in Nong Bua Lamphu province (5.45%) was statistically significantly lower than that of the south villages in Khon Kaen province (26.42%) (Fig 2B). When villages were grouped according to their proximity to waterbody types, infection prevalence did not vary much between villages located close to the reservoir and to the river inlets, but infection intensity was significantly higher for the reservoir villages at 93.72 epg than for the river villages at 38.54 epg.

**Fig 3:**
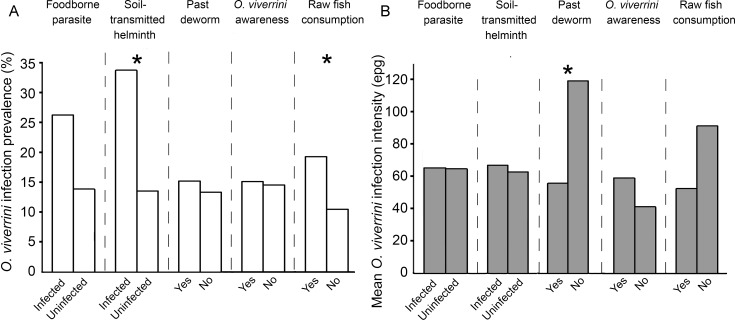
Other parasitic infections and human attitude and behavior as risk factors for *O*. *viverrini* infection. Association of (A) *O*. *viverrini* infection prevalence and (B) mean infection intensity with participants of other infections, deworming history, awareness, and raw fish consumption behavior. Significant differences in infection prevalence or intensity for bivariate analyses are indicated by the asterisks.

Bivariate analyses of the infection status of other foodborne parasites and soil-transmitted helminths revealed that *O*. *viverrini* infection prevalence was higher in participants who were also infected with other parasites, particularly those infected with soil-transmitted helminths (Fig 3A). Participants who had raw fish consumption behavior were found to be significantly associated with higher *O*. *viverrini* prevalence (Fig 3A), while the intensity of infection was significantly higher for participants who had not been dewormed (121.19 epg) than for those who had been (56.7 epg) (Fig 3B). However, such associations were not observed in the multivariate models. Past *O*. *viverrini* deworming history did not greatly influence *O*. *viverrini* infection prevalence (Fig 3A), and no statistically significant association was observed between *O*. *viverrini* awareness and both the *O*. *viverrini* infection prevalence and intensity.

**Fig 4:**
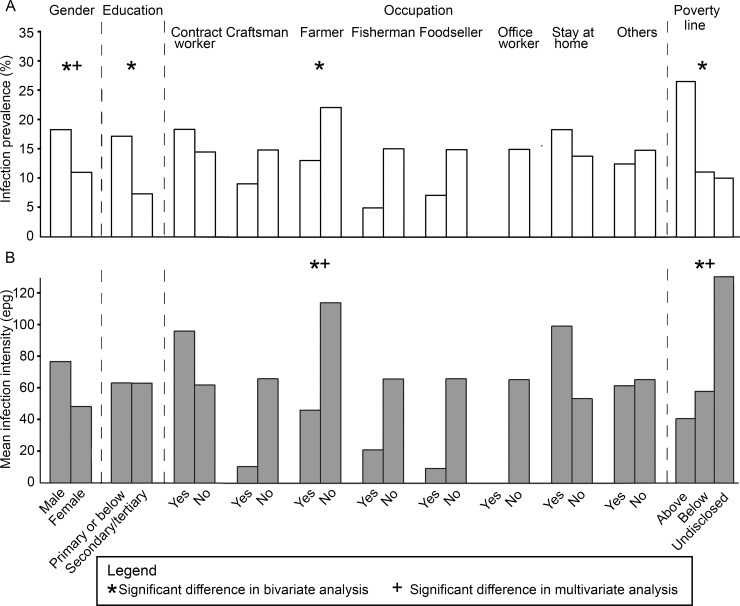
Socio-economic and demographic factors as risk factors for *O*. *viverrini* infection. (A) *O*. *viverrini* infection prevalence and (B) mean intensity of infected participants of various socio-economic and demographic factors.

The mean age of participants found infected with *O*. *viverrini* was 56.1 years while the mean age that of the uninfected was 52.0. Age was positively associated with infection prevalence in the multivariate model. Among social factors (Fig 4), both the bivariate and multivariate analyses suggested that gender was significantly associated with infection prevalence, while farming as an occupation and poverty line were significantly associated with infection intensity.

### Risk factors for infection prevalence and intensity and reasons for/for not consuming raw fish

Bivariate analyses indicated that the explanatory variables of the presence of soil-transmitted helminths, location, age, gender, education, farming as an occupation, above or below the poverty line, and raw fish consumption were significantly associated with *O*. *viverrini* infection prevalence (p < 0.05). Other foodborne parasitic infection was associated with *O*. *viverrini* infection prevalence at p < 0.20. These variables were hence entered into a multivariate regression model.

Results of the multivariate logistic regression model showed that the likelihood of infection was higher among villagers in the south, increased with age, and was the greatest in males and in those who consumed raw fish (Table 1). However, the consumption of raw fish is higher (63.6%) in the two villages located in the province of Nong Bua Lamphu (N-river and N-reservoir) when compared with the two villages studied in the province of Khon Kaen (S-river and S-reservoir) (52.2%) (χ^2^ = 7.31, df = 1, p-value = 0.01). Males were also more likely to consume raw fish (63.5%) than females (54.2%) (χ^2^ = 4.83, df = 1, p-value = 0.03). *O*. *viverrini* awareness was found to be negatively associated with raw fish consumption, with 28.6% of participants who were unaware of *O*. *viverrini* reported not eating raw fish as compared to 48.7% of participants who were aware of *O*. *viverrini* (χ^2^ = 7.86, df = 1, p-value = 0.01).

**Table 1:** Multivariate logistic regression model of infection prevalence. Only explanatory variables that are statistically significant in the model are displayed.

Variable			Adjusted OR	95% CI	p-value
*Spatial distribution*	Location				
		North	1.00		
		South	5.73	3.32–10.27	< 0.01
*Socio-economic/ demographic factors*	Age				
		> 21 years old	1.03	1.01–1.06	< 0.01
	Gender				
		Male	1.78	1.07–3.00	0.03
		Female	1.00		
*Attitudes and practices*	Raw fish consumption				
		Yes	2.71	1.57–4.83	< 0.01
		No	1.00		

OR, odds ratio; CI, confidence interval.

The variables of past *O*. *viverrini* deworming, waterbody type, farming as an occupation, and income relative to the poverty line were significantly associated with infection intensity (p < 0.05). Other foodborne parasitic infection and soil-transmitted helminth infection were associated with *O*. *viverrini* infection intensity at p < 0.20. Consequently, these variables were examined together in a multivariate Quasi-Poisson regression model. Results of the multivariate Quasi-Poisson regression model revealed that increased infection intensity was found in participants from villages located closest to the reservoir, participants who were not farmers, and participants who chose not to disclose their income information (Table 2). Comparison of spatial variation in human and fish infection indicated that, similar to human infection intensity, fish infection density was significantly higher in the reservoir waterbody type than the river waterbody type (t = 2.66, df = 10.22, p-value = 0.023), but not significantly different between the north and south (t = -0.04, df = 9.39, p-value = 0.97).

**Table 2:** Multivariate Quasi Poisson regression of infection intensity. Only explanatory variables that are statistically significant in the model are displayed.

Variable			Adjusted RR	95% CI	p-value
*Spatial distribution*	Waterbody				
		River	1.00		
		Reservoir	2.09	1.12–4.02	< 0.01
*Socio-economic/ demographic factors*	Occupation—Farmer				
		Yes	0.44		
		No	1.00	0.25–0.78	< 0.01
	Poverty line				
		Above	1		
		Below	1.02	0.48–2.19	0.96
		Undisclosed	2.71	1.34–5.63	< 0.01

RR, risk ratio; CI, confidence interval.

Participants who were unaware of *O*. *viverrini* were more than twice as likely to state that they ate raw fish because it tasted delicious, while those living in the S-river and S-reservoir villages were more likely to state that they ate it out of habit. The odds of eating raw fish because of friends decreased with every one-year increase in age. Males were also more than twice as likely to eat raw fish because of friends as females (Table 3).

**Table 3:** Factors associated with reasons for consumption among participants who consume raw fish dishes.

Reasons for consumption	Variable		Positive response (%)	OR	95% CI	p-value
*Delicious*	*O*. *viverrini* awareness					
		Yes	55.62	1		
		No	76.46	2.59	1.15–6.42	0.03
*Habit*	Location					
		North	27.27	1		
		South	50.88	2.76	1.68–4.59	< 0.01
*Eat with friends*	Age					
		> 21 years old	26.1	0.97	0.95–0.99	0.01
	Gender					
		Male	35	2.28	1.30–4.8	< 0.01
		Female	18.66	1		

OR, odds ratio; CI, confidence interval.

The likelihood of not eating raw fish in order to avoid being infected by *O*. *viverrini* was at least eight times higher among participants who were aware of the risks of infection. When asked about the reason for selecting the option of avoiding *O*. *viverrini* despite having responded “No” in the question regarding *O*. *viverrini* awareness, some of the participants explained that they had been encouraged by health volunteer workers or nurses to avoid eating raw fish because of the parasite, even though they were unsure about what the parasite was. Participants who knew about *O*. *viverrini* were also about seven times more likely to avoid eating raw fish due to other health reasons. Participants who did not know about *O*. *viverrini* were more likely to avoid eating raw fish because they dislike it. In addition, participants who said that they disliked raw fish were more likely not to have received treatment in the past, are younger, or live in either N-river or N-reservoir village (Table 4).

**Table 4:** Factors associated with reasons for non-consumption among participants who do not consume raw fish dishes.

Reasons for non-consumption	Variable		Positive response (%)	OR	95% CI	p-value
*Avoid O*. *viverrini*	*O*. *viverrini* awareness					
		Yes	71.63	8.04	4.19–16.80	< 0.01
		No	13.33	1		
*Avoid due to other health reasons*	*O*. *viverrini* awareness					
		Yes	26.67	6.96	1.62–27.08	< 0.01
		No	4.96	1		
*Dislike*	Past *O*. *viverrini* deworm					
		No	41.82	2.78	1.05–8.41	0.05
		Yes	14.63			
	Location					
		North	41.05	2.63	1.11–6.70	0.03
		South	25.29	1		
	Age					
		> 21 years old	33.89	0.97	0.93–0.99	0.03
	*O*. *viverrini* awareness					
		Yes	24.82	1		
		No	53.33	3.19	0.91–11.53	0.07

OR, odds ratio; CI, confidence interval.

### Healthcare focus, perceptions, and chemotherapy history

In the S-river village, there have been no attempts to determine *O*. *viverrini* infections, including fecal examination, for at least 10 years. The priorities of the health center of the S-river village focused on the health effects of pesticide use and respiratory tract infections (Table 5). By comparison, in the S-reservoir village, fecal tests of *O*. *viverrini* infection were performed in 2007 and 2008, with infection prevalence estimated at 0% and 2%, respectively (Table 5). Because of funding constraints, the direct smear technique was employed and only relatively few people were tested in the village. A recent fecal examination done in 2014, in villages belonging to the same health jurisdiction as the S-reservoir village, yielded infection prevalence similar to that of the S-reservoir village in 2007 and 2008. Similar to the S-river village, the S-reservoir health center staff did not view opisthorchiasis as a top priority; instead, diabetes and hypertension were the main concerns.

**Table 5:** Summary of key findings on health centers’ chemotherapy efforts and current top health concerns by villages. Only infection prevalence was collected by the health centers, not infection intensity.

Village	Past chemotherapy	Diagnostic tool	*O*. *viverrini* prevalence	Top health concerns
*S-river*	None in the past 10 years	-	-	Toxicity from pesticides and respiratory tract infections
*S-reservoir*	2007	Direct smear	0%	Diabetes and hypertension
	2008	Direct smear	2%	
	2014*	Direct smear	0.25%	
*N-river*	2011	Single Kato-Katz thick smear	5.25%	Teenage pregnancy and parasitic diseases
	2012	Single Kato-Katz thick smear	2.26%	
*N-reservoir*	2012	Single Kato-Katz thick smear	8.24%	Diabetes, hypertension, work related injuries, and gastrointestinal diseases
	2013	Single Kato-Katz thick smear	0.27%	

* The 2014 examination was conducted for other villages in the same sub-district of the S-reservoir village.

In the N-river village, fecal examination was performed in 2011 and 2012 with infection prevalence estimated at 5.25% and 2.26%, respectively. The local health center prioritized teenage pregnancy and parasitic diseases as the top health concerns, with campaigns aimed at reducing rates of teenage pregnancy organized by health center staff. In the N-reservoir village, fecal examination was performed in 2012 and 2013, and *O*. *viverrini* prevalence was estimated at 8.24% and 0.27%, respectively. Different from the south villages, the local health centers of both N-reservoir and N-river villages used the single Kato-Katz thick smear technique for *O*. *viverrini* infection test. Hypertension and diabetes, the top health concerns of the S-reservoir village, were also prioritized by the local health center staff of the N-reservoir village as the major health concerns, among work related injuries and gastrointestinal diseases (Table 5).

## Discussion

This study revealed that *O*. *viverrini* infection intensity is associated with the proximity of villages to waterbodies (rivers versus the reservoir), farming as an occupation, and income level relative to the poverty line. Infection prevalence is associated with village location (villages to the north of the reservoir versus the south), age, gender, and raw fish consumption. Among the factors influencing the consumption of raw fish, the most notable is the association between males and the practice of consuming raw fish along with friends in social gatherings. Among the factors associated with the non-consumption of raw fish, the most notable observation is the association between *O*. *viverrini* awareness and the avoidance of raw fish consumption for disease prevention or health issues. The physical environment, namely proximity to different kinds of waterbodies, and the social environment including socio-economic, demographic, and behavioral factors, thus influence *O*. *viverrini* infection differently in infection intensity and prevalence. Additionally, health center focus affects the risk perceptions, funding, and infection prevalence among the local population.

### Waterbodies as a contributing factor to the pathogenic landscape for *O*. *viverrini* infection

The results show that examining prevalence alone risks ignoring important parasitic infection trends. Although there was not a significant difference in *O*. *viverrini* infection prevalence between villages located near river inlets as compared with villages near the Ubolratana reservoir, infected villagers from near the reservoir had more than double the parasite intensity as compared with villagers from near the river. The pattern of infection intensities among humans thus matched the infection density of fish collected from these locations, with higher overall fish infection associated with the reservoir when compared with river inlets [42], while there was no difference in infection density in the fish from the south or north reservoir.

Distance to waterbody had an impact on where villagers tended to source the fish used in raw fish dishes; villagers who lived close to the river tended to procure fish from the river, while those living close to the reservoir tended to procure fish from the reservoir. As fish in the reservoir is more plentiful, people who lived farther away from both river inlets and reservoir also tended to rely upon fish caught from the reservoir [42]. Differences in fish infection levels depending on waterbody can affect the level of exposure of humans to the risk of infection, as is evident in the results; the average intensity of infection in the S-river village was low despite the lack of chemotherapy effort, and lower than both N-reservoir and S-reservoir villages, despite the recent chemotherapy treatment efforts in the N-reservoir village in particular.

Using only infection prevalence as the measure of success for intervention effort can problematically lead to individuals with high infection intensities in low prevalence areas being overlooked. Even in individuals with low infection intensity, it is possible to develop CCA, as observed in this study and other biomedical studies. During the course of the survey in this study, a participant who was tested negative for infection was diagnosed with CCA and passed away shortly after diagnosis. The participant had a history of raw fish consumption and no record of past *O*. *viverrini* treatment. The apparent absence of *O*. *viverrini* eggs in the fecal sample could have been due to a low intensity of infection or bile duct obstruction [48]. Biomedical studies show that opisthorchiasis-induced inflammation can lead to the development of *O*. *viverrini*-induced advanced periductal fibrosis (APF) and CCA, which are driven by common cellular mechanisms, marked by elevated level of plasma interleukin-6 [49]. Participants with the most elevated level of plasma interleukin-6 were found to have an increased risk of 19 and 150 times of developing APF and CCA, respectively, as compared with other *O*. *viverrini* infected individuals with no detectable plasma interleukin-6 [49]. The risk of developing APF was found to increase with increased infection intensity [50,51] and duration of infection [50].

The findings in this study are of relevance to the concept of One Health, as they highlight the close relationship between the health of humans and that of the health/infection status of the animal hosts and physical environment. The findings identify the reservoir as an important target for opisthorchiasis intervention efforts and also underscore the importance of considering infection intensity in the understanding of the pathways through which the parasite is transmitted. Comparative multilocality studies are necessary to gain useful insights into the similarity or difference in relationships between opisthorchiasis and the environment in such reservoir systems.

### Socio-economic, demographic, and behavioral influencing factors

Far higher infection prevalence in males than in females accords with findings from some previous studies [52,53]. Little difference in prevalence between genders has also been reported [38,54], although Hasewell-Elkin et al [54] notes that the frequency of high infection intensities may be higher among males. Males are also more likely to die from opisthorchiasis. As males are often the main income earners in families in Thailand, opisthorchiasis can exert a disproportionate economic toll on those affected [49]. One reason for a higher infection prevalence and intensity among males is likely to be their socializing behavior: raw fish dishes are often available for consumption at social gatherings of males. Infection prevalence also tended to increase with age from 21 years in this study. This finding is at odds with existing results, which indicate a plateauing of infection prevalence in the late teens followed by a decline in later life [34,35].

In some studies, fishermen and/or farmers were found to have higher infection prevalence [38,45]. This is because local fishermen often make a dish of Koi pla from their catch to celebrate that day’s fishing [23]. Farmers may also harvest fish from their rice paddies and prepare and consume the catch on the spot. Conversely, in this study, infection in fishermen and farmers was not significantly higher than for other occupations. Higher infection intensities were found only in participants who were not farmers. The participants who were not farmers have other occupations including contract worker, craftsman, fisherman, foodseller, office worker, stay at home, and others. There was however no significant difference in intensity among people who belonged to those occupation types and those who do not (Fig 4), suggesting that the observed higher infection intensity in people who are not farmers is not determined by a single occupation type. Higher infection intensity was also found only in participants who chose not to disclose their income information. No clear pattern was observed between occupation types and the disclosure of income (S2 Table). Consequently, the socio-economic and demographic factors selected in this study could not identify the specific groups of people at risk of higher infection intensity. Recent chemotherapy efforts in three of the four villages may have weakened links with the range of factors that result in infections.

While there was no significant difference in infection prevalence and intensity with *O*. *viverrini* awareness, *O*. *viverrini* awareness appeared to reduce the proportion of people who reported consuming raw fish. Participants who were aware of *O*. *viverrini* were also more likely to avoid raw fish consumption in order to avoid opisthorchiasis and other health issues, while participants who were unaware of *O*. *viverrini* were more likely to avoid consumption due to personal dietary preferences. Awareness campaigns may be able to affect personal health decisions to a certain extent, although more holistic effort is needed to tackle this long-standing issue.

### Variation in efficacy of health center focus

The pattern of villagers residing in the south of the reservoir being much more likely to be infected than villagers in the north may reflect inter-provincial differences in health priorities and treatment efforts. Use of praziquantel to treat infections can result in a sharp decline in prevalence [55]. For example, praziquantel administration brought about an immediate decline of *O*. *viverrini* prevalence from approximately 60% to 14%, while infections among the control, untreated group increased from 65% to 71% within the same time frame [56]. Likewise, a similar decline in prevalence (67% to 16%) during three years of praziquantel administration is reported in Sripa et al [37]. Favorable results following chemotherapy-based treatment efforts do not necessarily imply long-term success of a campaign, however. Resurgence of infection has been observed soon after the cessation of a campaign [57].

This study revealed disparate healthcare concerns and opisthorchiasis control efforts. While the particular focus of the health center can be tailored to the needs of the villages within the sub-district [58], funding allocation for healthcare is decided at provincial level. As the S-river and S-reservoir villages are part of districts in Khon Kaen province, where the Isarn Agenda was not implemented, limited funding was made available for opisthorchiasis control efforts.

The lack of fecal examination for *O*. *viverrini* infection for the past decade may account for the high infection prevalence recorded in the S-river village. In the S-reservoir village, where a relatively limited treatment program was in place, infection prevalence was second only to the S-river village. Direct smear was used in both villages to test for infections as it is the most affordable, despite it being the least sensitive method [59]. The low sensitivity of the test may have led to erroneous results in the form of low prevalence data. Due to an apparent low prevalence of *O*. *viverrini* and increasing prevalence in chronic diseases, particularly diabetes and hypertension, it is not unexpected that the local health center staff increasingly prioritize such chronic diseases as their top health concerns. Coupled with the affordability and simplicity of testing for diabetes and hypertension, regular blood sugar tests and blood pressure tests are offered by the health centers, which may in turn shift the health focus of the villagers to such chronic diseases. Indeed, during the course of this study, the villagers and health center staff of the south villages have expressed that *O*. *viverrini* infection is not an issue of concern in the village. Coincidentally, fecal examinations were carried out by the health center staff in 2014, the same year of this present study, to survey *O*. *viverrini* infections in villages within the sub-district of the S-reservoir village. As the health center knew about our intent of sampling in the S-reservoir village, the health center sampling was conducted in all villages of the sub-district except the S-reservoir village. Their survey reported an overall infection prevalence of 0.25% for those villages, which was close to the prevalence observed for the S-reservoir village in 2007 and 2008 (Table 5). Nevertheless, the prevalence was in stark contrast to the much higher levels obtained in this study (i.e., 18.45% for the S-reservoir and 40.21% for the S-river, Fig 3A), with the disparity likely due to the difference in sensitivity of fecal examination methods employed. Unfortunately, disparate healthcare focus, coupled with limited funding and a less sensitive opisthorchiasis screening method may have given villagers–and health center staff–a false impression of the importance of opisthorchiasis. Villagers who consume raw fish may be lulled into a false sense of security when any tests for *O*. *viverrini* infection generate negative results, despite the frequent consumption of raw fish, as mentioned by several villagers interviewed.

Fecal examinations were carried out by the health centers concerned on a greater number of individuals in the N-river and N-reservoir villages. The diagnostic tests for *O*. *viverrini* infection also relied upon the more sensitive single Kato-Katz thick smear. Infection prevalence in the N-river village, at 5.3% and 2.3% in, respectively, 2011 and 2012, was close to the 4.6% prevalence obtained in this study. Similarly, close results were found for the N-reservoir village (8.2% and 0.3% in, respectively, 2012 and 2013, compared with 6.4% in this study). Despite the increased focus on opisthorchiasis and CCA after the implementation of Isarn Agenda, the lower infection prevalence, and no significant differences in awareness of the risks of *O*. *viverrini* infection, the proportion of participants who reported eating raw fish remained high in these two north villages.

This study emphasizes the influence of health center focus on *O*. *viverrini* infection prevalence. While most of the prior work has emphasized on human behavior and social risk factors for helminth diseases including opisthorchiasis, healthcare focus and provision can greatly affect the risk of infection and the vulnerability of local populations [60]. Healthcare focus and provision can also sheds light on the varying stakeholders’ values determining the pathogenic landscape of diseases. Stakeholders’ values can influence the outcome and direction of healthcare provision as illustrated in the variation in provincial funding and health center focus in this study. In the cases of other disease intervention efforts that substantially rely on external donor funding, there can be potential conflicting interests between local population, funding donors, or even pharmaceutical companies [61]. The influences of healthcare focus and interests of other stakeholders thus need to be considered when deciphering the factors contributing to disease risks.

The holistic approach in this study has identified important features of helminth parasitism, specifically, opisthorchiasis, which include the connectivity of animal hosts and humans facilitated by waterbodies and human behavior; human behavioral and physical environmental conditions that facilitated reinfection; and the influence of healthcare interventions on infection prevalence. Identification of such features of parasitism is an important contribution to the framework of One Health approach [23], where consideration of helminth diseases has largely been overlooked [22].

## Conclusion

While the role of socio-economic, demographic, and behavioral risk factors on *O*. *viverrini* infection have been investigated in previous studies, this study identified other additional influential environmental and healthcare implementation risk factors in *O*. *viverrini* infection. Humans interact with the environment reciprocally, thereby influencing their risks of disease infection. Human modifications of the environment, particularly in the form of dam construction and reservoir creation, have changed the aquatic habitats for the *O*. *viverrini* intermediate fish hosts. As *O*. *viverrini* infection intensities in the fish vary across different waterbody types, humans affect their risks of consuming *O*. *viverrini* infected raw fish through fish procurement location preferences. In opisthorchiasis studies and that of other helminthiasis, infection intensity is still much less frequently reported. The importance of considering infection intensity in a cross-sectional infection study is exemplified in this study, owing to the critical role of intensity in the most serious forms of many infectious diseases, including opisthorchiasis, and in providing insights into parasite transmission risks.

Healthcare focus can directly affect human infection prevalence through chemotherapy and indirectly guide villagers’ risk perceptions through the choices of health campaigns or monitoring programs. Chemotherapy in the case of helminthiases such as opisthorchiasis is only palliative, with re-infections quickly occurring if the underlying factors that expose humans to infection are not dealt with. There is thus a need for a holistic approach to integrate the factors accounting for the broader pathogenic landscape within which diseases such as opisthorchiasis persist.

## Supporting Information

S1 Table Summary of variables. Summary of variables examined in this study, their possible outcomes, and sources.
(DOCX)

S2 Table Proportion of participants with income disclosed or undisclosed by occupation types. Disclosed income was listed by above or below poverty line.
(DOCX)

S1 File Participant Questionnaire in English. The participant questionnaire in English was translated into Thai to be used for data collection.
(PDF)

S2 File Participant Questionnaire in Thai. The participant questionnaire in Thai was used for data collection.
(PDF)

S3 File Descriptive statistics of dataset. Data are classified according to villages.
(XLSX)

S1 Checklist STROBE checklist. (DOC)
