# Supplementary material for: Uncovering the Pathogenic Landscape of Helminth (Opisthorchis viverrini) Infections: A Cross-Sectional Study on Contributions of Physical and Social Environment and Healthcare Interventions
Source: PLoS Negl Trop Dis. 2016 Dec 7;10(12):e0005175. doi: 10.1371/journal.pntd.0005175 (PMC5142777; doi:10.1371/journal.pntd.0005175)
Supplement: S1 Table — Summary of variables examined in this study, their possible outcomes, and sources. (DOCX) [file pntd.0005175.s001.docx]

**S1 Table. Summary of variables.** Summary of variables examined in this study, their possible outcomes, and sources.

| **Variable examined** | **Possible outcome** | **Source of variable** |
| --- | --- | --- |
| Location of villages | North or South | Study design of this study |
| Villages proximity to waterbody | River or Reservoir | Study design of this study |
| Fish infection by location | North or South | Ong et al [42] |
| Fish infection by waterbody | River or Reservoir | Ong et al [42] |
| Foodborne parasite | Yes or No | Human infection survey of this study |
| Soil-transmitted helminth | Yes or No | Human infection survey of this study |
| Past deworm (past treatment of *O. viverrini*) | Yes or No | Questionnaire of this study and records from the health centers, obtained for this study |
| *O. viverrini* awareness | Yes or No | Questionnaire of this study |
| Raw fish consumption | Yes due to (a) Delicious, (b) Habit, (c) Eat with friends, or (d) Take deworming medicine; or No due to (a) Avoid *O. viverrini*, (b) Other health reasons, or (c) Dislike raw fish | Questionnaire of this study |
| Gender | Male or Female | Questionnaire of this study |
| Education | Primary or below; or Secondary/tertiary | Questionnaire of this study |
| Occupation-Contract worker | Yes or No | Questionnaire of this study |
| Occupation-Craftsman | Yes or No | Questionnaire of this study |
| Occupation-Farmer | Yes or No | Questionnaire of this study |
| Occupation-Fisherman | Yes or No | Questionnaire of this study |
| Occupation-Foodseller | Yes or No | Questionnaire of this study |
| Occupation-Office worker | Yes or No | Questionnaire of this study |
| Occupation-Stay at home | Yes or No | Questionnaire of this study |
| Occupation-Others | Yes or No | Questionnaire of this study |
| Poverty line | Above, Below, or Undisclosed | Questionnaire of this study |
| Past chemotherapy efforts by health center | Not applicable | Records from the health centers, obtained for this study |
| Diagnostic tool used | Not applicable | Records from the health centers, obtained for this study |
| Past *O. viverrini* prevalence | Not applicable | Records from the health centers, obtained for this study |
| Top health concerns of health center | Not applicable | Records from the health centers, obtained for this study |
