# Supplementary material for: Uncovering the Pathogenic Landscape of Helminth (Opisthorchis viverrini) Infections: A Cross-Sectional Study on Contributions of Physical and Social Environment and Healthcare Interventions
Source: PLoS Negl Trop Dis. 2016 Dec 7;10(12):e0005175. doi: 10.1371/journal.pntd.0005175 (PMC5142777; doi:10.1371/journal.pntd.0005175)
Supplement: S2 Table — Disclosed income was listed by above or below poverty line. (DOCX) [file pntd.0005175.s002.docx]

**S2 Table. Proportion of participants with income disclosed or undisclosed by occupation types.** Disclosed income was separated by above or below poverty line.

| **Occupation types** | **Income disclosed (%)** | | **Undisclosed (%)** |
| --- | --- | --- | --- |
|  | **Above poverty line** | **Below poverty line** |  |
| Contract worker | 18 | 53 | 29 |
| Craftsman | 9 | 64 | 27 |
| Farmer | 22 | 53 | 25 |
| Fisherman | 30 | 50 | 20 |
| Foodseller | 21 | 36 | 43 |
| Office worker | 62 | 15 | 23 |
| Stay at home | 24 | 53 | 23 |
| Others | 13 | 75 | 13 |
