# Supplementary material for: Uncovering the Pathogenic Landscape of Helminth (Opisthorchis viverrini) Infections: A Cross-Sectional Study on Contributions of Physical and Social Environment and Healthcare Interventions
Source: PLoS Negl Trop Dis. 2016 Dec 7;10(12):e0005175. doi: 10.1371/journal.pntd.0005175 (PMC5142777; doi:10.1371/journal.pntd.0005175)
Supplement: S1 File — The participant questionnaire in English was translated into Thai to be used for data collection. (PDF) [file pntd.0005175.s003.pdf]

**Participant Questionnaire**

**Landscape and human behavior factors determining fish to human transmission of liver fluke, *O. viverrini*, around the Ubolratana reservoir, northeast Thailand.**

**Instruction:** Fill in the blanks. Please check the appropriate boxes with a ✓.

## Participant information

1. What is your relationship to the head of the household? Please tick.

- ☐ Head of household
- ☐ Grandfather/Grandmother
- ☐ Father/Mother
- ☐ Uncle/Aunt
- ☐ Brother/Sister
- ☐ Cousins
- ☐ Son/Daughter
- ☐ Nephew/Niece
- ☐ Grandson/Granddaughter

2a. When was the last time you took the deworming drug for liver fluke? Please tick.

- ☐ Never **(skip 2b and 2c)**
- ☐ One year ago
- ☐ A few years ago
- ☐ More than 10 years ago

2b. Did you have a fecal examination to test for the parasite before taking the drug? (circle) Yes/No

2c. Where did you get for medicine from? Please tick.

- ☐ From health center
- ☐ From hospital
- ☐ From friends/relatives
- ☐ Others:______________________________________

3. Occupation (may choose more than 1 option)

- ☐ Housewife
- ☐ Farmer
- ☐ Fisherman
- ☐ Contract worker
- ☐ Retiree
- ☐ Food seller
- ☐ Craftsman
- ☐ Office work
- ☐ Student
- ☐ Others:________

4. Education level. Please tick.

- ☐ Illiterate
- ☐ Primary
- ☐ Secondary
- ☐ Diploma
- ☐ University
- ☐ Others:____________

5. Personal average income/month

______________________________

6. Household income/month

______________________________

7. Number of people in household

______________________________

**<u>Fish consumption behaviour</u>**

8. Do you eat “koi pla” , “pla som”, “pla ra” or “mum”? How often?
Do not eat: **0**
Daily: **1**
Weekly: **2**
Monthly: **3**
Special occasions/ once a year: **4**

| | Cooked frequency | Raw frequency |
|---|---|---|
| Koi pla | | |
| Pla som | | |
| Pla ra | | |
| Mum | | |

*Indicate if the fish used are not cyprinids.

9. Do you know about *O.viverrini* parasite? Please tick.

- ☐ Yes
- ☐ No

**If respondent eats raw koi pla, pla som or mum (exclude pla ra):**
10a. Why do you choose to eat raw fish (koi pla/ pla som/mum)?
(may choose more than 1 option)

- ☐ Delicious
- ☐ Take medicine
- ☐ Eat with friends
- ☐ Habit
- ☐ Others:________________

**If respondent do not eat raw koi pla or pla som (exclude pla ra):**

10b. Why do you not eat raw fish (koi pla/ pla som/mum)? Please tick.

- ☐ Avoid OV
- ☐ Avoid raw fish due to other health reasons eg. Stomachache
- ☐ Do not like raw fish
- ☐ Others:__________________

11. What are your most commonly eaten types of fish (cooked and raw)? How often do you eat them?

Do not eat: **0**
Daily: **1**
Weekly: **2**
Monthly: **3**
Special occasions/ once a year: **4**

| | Consumption frequency |
|---|---|
| Cyprinid fish | |
| Catfish | |
| Snakehead | |
| Others:_________________ | |

……………………………..

End of Questionnaire

Thank You
