# Supplementary material for: Uncovering the Pathogenic Landscape of Helminth (Opisthorchis viverrini) Infections: A Cross-Sectional Study on Contributions of Physical and Social Environment and Healthcare Interventions
Source: PLoS Negl Trop Dis. 2016 Dec 7;10(12):e0005175. doi: 10.1371/journal.pntd.0005175 (PMC5142777; doi:10.1371/journal.pntd.0005175)
Supplement: S2 File — The participant questionnaire in Thai was used for data collection. (PDF) [file pntd.0005175.s004.pdf]

แบบสอบถามการเคลื่อนย้ายและพฤติกรรมการกินปลาของประชาชน

คำชี้แจง เติมข้อความลงในช่องว่างให้สมบูรณ์ และทำเครื่องหมายในช่องว่าง ✓ หน้าคำตอบที่ท่านต้องการ

ส่วนที่ ข้อมูลทั่วไป 1

1. ท่านมีความสัมพันธ์ใดกับเจ้าของบ้าน กรุณาเลือก

☐ เจ้าของบ้าน ☐ พี่/ น้อง

☐ สามี/ภรรยา ☐ ปู่ตา/ ย่ายาย

☐ พ่อ/ แม่ ☐ ลูกพี่ลูกน้อง

☐ ลูกชาย/ ลูกสาว ☐ ลุง/ ป้า

☐ หลายชาย/ หลานสาว (เป็นลูกของพี่หรือน้อง)

☐ หลายชาย/ หลานสาว (เป็นลูกของลูก)

2. ครั้งสุดท้ายที่ท่านกินยาถ่ายพยาธิสำหรับรักษาโรคพยาธิใม้ตับ

☐ ไม่เคย (ข้ามข้อ 2ข และ 2ค)

☐ น้อยกว่า 1 ปี

☐ มากกว่า 1 ปี

☐ มากกว่า 10 ปี

2ข. ท่านได้ตรวจอุจจาระก่อนกินยาถ่ายพยาธิหรือไม่ (วงกลมคำตอบ)

ใช่ / ไม่ใช่

2ค. ท่านได้รับยาถ่ายพยาธิจากที่ใด

☐ จากสถานีอนามัย

☐ จากโรงพยาบาล

☐ จากเพื่อน/ญาติพี่น้อง

☐ อื่นๆ

3. อาชีพ (ตอบได้มากกว่า 1ข้อ)

- ☐ 1.แม่บ้าน
- ☐ 2.ทำไร่/ทำนา
- ☐ 3.ชาวประมง
- ☐ 4.ลูกจ้าง
- ☐ 5.ไม่ได้ทำงาน
- ☐ 6.ขายอาหาร
- ☐ 7.ช่างฝีมือ
- ☐ 8.ข้าราชการ/รัฐวิสาหกิจ
- ☐ 9.นักเรียน
- ☐ 10.อื่นๆ

4. จบการศึกษาระดับใด

- ☐ 1. อ่านไม่ออก
- ☐ 2. ประถมศึกษา
- ☐ 3. มัธยมศึกษา
- ☐ 4. ปวช/ปวส
- ☐ 5.ปริญญาตรี
- ☐ 6.อื่นๆ

5. รายได้เฉลี่ยต่อเดือน ……….......บาท

6. รายได้ของครอบครัวเฉลี่ยต่อเดือน ..…………..บาท

7. จำนวนสมาชิกในครัวเรือน …..……..…..คน

ส่วนที่ 2 พฤติกรรมการกินปลา

8. ท่านเคยกินอาหารต่อไปนี้หรือไม่ อย่างไร

ไม่เคยกิน ใส่ =0 กินทุกวัน ใส่ =1

กินบ่อยๆทุกสัปดาห์ ใส่ =2 กินบางครั้งทุกเดือน ใส่ =3

นานๆครั้ง เฉพาะโอกาสสำคัญๆของปี ใส่=4

	ปรุงสุก	กินดิบ หรือสุกๆดิบๆ
ก้อยปลา		
ปลาส้ม/ส้มปลา		
ปลาร้า		
หม่ำ		

*ระบุชนิดปลาด้วย ถ้าไม่ใช่ปลาขาวปลาตะเพียน Indicate if the fish used are not cyprinids.

9. ท่านรู้จักพยาธิใบไม้ตับหรือไม่

- ☐ 1.รู้จัก
- ☐ 2. ไม่รู้จัก

10 คำถามเกี่ยวกับการกินก้อยปลาดิบ ปลาส้มดิบ หรือหม่ำดิบ

10a. ถ้าอาสาสมัครตอบว่าเคยกินก้อยปลาดิบ ปลาส้มดิบ หรือหม่ำดิบ (ไม่รวมปลาร้าดิบ)

ทำไมท่านจึงกินก้อยปลาดิบ ปลาส้มดิบ หรือหม่ำดิบ (เลือกได้มากกว่า 1 คำตอบ)

☐ 1.อร่อย

☐ 2.กินยารักษาพยาธิแล้ว

☐ 3.กินกับเพื่อนๆ

☐ 4.เป็นความเคยชิน

☐ 5.อื่นๆ _______________

10b. ถ้าอาสาสมัครตอบว่าไม่เคยกินก้อยปลาดิบ ปลาส้มดิบ หรือหม่ำดิบ (ไม่รวมปลาร้าดิบ)

ทำไมท่านจึงไม่กินก้อยปลาดิบ ปลาส้มดิบ หรือหม่ำดิบ

☐ หลีกเลี่ยงการติดเชื้อพยาธิใบไม้ตับ

☐ หลีกเลี่ยงจากปัญหาด้านสุขภาพ เช่น ปวดท้อง

☐ ไม่ชอบกินปลาดิบ

☐ อื่นๆ __________________

11. ปลาชนิดใดต่อไปนี้ ที่ท่านกิน (ทั้งปรุงสุกและกินดิบ) และบ่อยครั้งเพียงใด

ไม่เคยกิน	ใส่ =0
กินทุกวัน	ใส่ =1
กินบ่อยๆทุกสัปดาห์	ใส่ =2
กินบางครั้งทุกเดือน	ใส่ =3
นานๆครั้ง เฉพาะโอกาสสำคัญๆของปี	ใส่ =4

	ความถี่ของการกิน
11a.ปลาขาวปลาตะเพียน	
11b. ปลาดุก	
11c. ปลาช่อน	
11d. อื่นๆ	

....

ขอขอบคุณ
